# No evidence that crayfish carcasses produce detectable environmental DNA (eDNA) in a stream enclosure experiment

**DOI:** 10.7717/peerj.9333

**Published:** 2020-06-11

**Authors:** Amanda N. Curtis, Eric R. Larson

**Affiliations:** 1 Program in Ecology, Evolution, and Conservation Biology, University of Illinois at Urbana-Champaign, Urbana, IL, United States of America; 2 Department of Natural Resources and Environmental Sciences, University of Illinois at Urbana-Champaign, Urbana, IL, United States of America

**Keywords:** Invasive species, Endangered species, *Procambarus clarkii*, Red swamp crayfish, Wildlife management

## Abstract

Environmental DNA (eDNA) is an emerging tool for monitoring invasive and imperiled species, particularly at low densities. However, the factors that control eDNA production, transport, and persistence in aquatic systems remain poorly understood. For example, the extent to which carcasses produce detectable eDNA is unknown. If positive detections are associated with dead organisms, this could confound monitoring for imperiled or invasive species. Here, we present results from one of the first studies to examine carcass eDNA in situ by deploying carcasses of the invasive red swamp crayfish (*Procambarus clarkii*) in a stream enclosure experiment for 28 days. We predicted that carcasses would initially produce eDNA that would decline over time as carcasses decayed. Unsurprisingly, crayfish carcasses lost biomass over time, but at the conclusion of our experiment much of the carapace and chelae remained. However, no eDNA of *P. clarkii* was detected in any of our samples at the crayfish density (15 *P. clarkii* carcasses at ∼615 g of biomass initially), stream flow (520–20,319 L/s), or temperature (∼14–25 °C) at our site. Subsequent analyses demonstrated that these results were not the consequence of PCR inhibition in our field samples, poor performance of the eDNA assay for intraspecific genetic diversity within *P. clarkii*, or due to the preservation and extraction procedure used*.* Therefore, our results suggest that when crayfish are relatively rare, such as in cases of new invasive populations or endangered species, carcasses may not produce detectable eDNA. In such scenarios, positive detections from field studies may be more confidently attributed to the presence of live organisms. We recommend that future studies should explore how biomass, flow, and differences in system (lentic vs. lotic) influence the ability to detect eDNA from carcasses.

## Introduction

Since the first application of environmental DNA (eDNA) to macrobiota (Ficetola et al., 2008), this methodology has emerged as an important conservation tool capable of detecting invasive and imperiled species at low abundances (e.g., Foote et al., 2012; Dougherty et al., 2016). Although eDNA has successfully been applied to a variety of environments (soil, freshwater, marine) and organisms (e.g., Jerde et al., 2011; Yoccoz et al., 2012), factors that control eDNA production, transport, and persistence remain poorly understood (Barnes & Turner, 2016). Therefore, it is imperative to understand which factors are important to eDNA detection to use this tool reliably for conservation and management.

A positive detection of eDNA does not necessarily indicate that the target organism is currently present in the system at the time of sampling. Detection may indicate the organism was present in the past (buried eDNA), eDNA may have been transported from elsewhere, or detection may have been caused by field or laboratory contamination. Moreover, eDNA could have been produced by a carcass and thus not indicate the presence of a live target organism (Roussel et al., 2015). False positives arising from a variety of sources can be problematic for management of either rare native or invasive species. Thus, it is important to know whether positive detections indicate that the target species is currently present, or whether the organism is no longer in that area or dead. In instances where eDNA is used to monitor reintroductions or translocations of imperiled species (e.g., Cowart et al., 2018a), it is necessary to know whether positive detections truly indicate the presence of live organisms, or whether carcasses from a failed management action are producing detectable eDNA. Alternatively, if eDNA is used to assess the success of an invasive species removal effort, false positives from carcasses could result in unnecessary and costly additional removal efforts (e.g., Merkes et al., 2014; Carim et al., 2020). Therefore, understanding whether carcasses produce eDNA, and under what contexts, is necessary to successfully employ eDNA as a management tool.

Research examining whether carcasses can produce detectable eDNA has been limited (Merkes et al., 2014; Kamoroff & Goldberg, 2018; Tillotson et al., 2018). Tillotson et al. (2018) found that decaying fish carcasses released more eDNA than living fish during sockeye salmon (*Oncorhynchus nerka*) spawning. Similarly, using a chamber experiment with silver carp (*Hypophthalmichthys molitrix*), Merkes et al. (2014) recovered high levels (22 million copies/L) of eDNA from carcasses for up to 28 days and found that carcass biomass was positively related to eDNA in water samples. Alternatively, Kamoroff & Goldberg (2018) found that goldfish (*Carassius auratus*) carcasses produced detectable eDNA for weeks post-mortality, but positive detections only came from water samples collected at the bottom of the water column near carcasses (here in a 2 L container). While previous research indicates that carcasses are capable of producing detectable eDNA, these studies (Merkes et al., 2014; Kamoroff & Goldberg, 2018) have been largely been confined to microcosm or laboratory studies that represent lentic (standing water) conditions, and consequently there is a need to examine these relationships in situ, in lotic (flowing water) environments, and at low abundances.

Our purpose was to examine whether decaying crayfish carcasses in a stream under natural conditions produce detectable eDNA. We chose to use crayfish because there are a number of invasive and imperiled crayfish that are currently targets of management actions (removal of invasive species and reintroductions of rare species), many of which use eDNA to monitor populations (e.g., Cai et al., 2017; Larson et al., 2017; Cowart et al., 2018a; Rice, Larson & Taylor, 2018; Robinson, De Leaniz & Consuegra, 2019). Our study organism, red swamp crayfish (*Procambarus clarkii)*, is native to the southern United States of America (USA) and northeastern Mexico, but is globally invasive (Huner, 2002; Oficialdegui et al., 2019). *Procambarus clarkii* has been associated with declines in macrophytes, macroinvertebrates, amphibians, fish, and native crayfish (Gherardi, 2006; Twardochleb, Olden & Larson, 2013). A population of *P. clarkii* has recently established in the Chicago River in Illinois, USA (Taylor & Tucker, 2005; Peters et al., 2014), where it has been a target of research and eradication efforts (O’Shaughnessey & Keller, 2019). In this study, we deployed dead *P. clarkii* (removed from the Chicago River) in a stream enclosure experiment and collected water samples to examine relationships between the decay of crayfish carcasses and detection of eDNA. We expected that crayfish carcasses would release eDNA, and that the amount of eDNA detected would decline over time in response to the loss of carcass biomass. Because eDNA can persist in water ≤44 days after organisms have been removed (Dejean et al., 2011; Thomsen et al., 2011; Goldberg et al., 2013), we also examined if eDNA could be detected after crayfish carcasses were removed from the stream. Our study has important implications for monitoring of both invasive and imperiled crayfish using eDNA, as well as for other organisms in which carcasses could contribute to positive eDNA detections.

## Materials & Methods

### Study site

We conducted our study in the Saline Branch of the Vermilion River (40°07′44″N, 88°09′05″W) located within the Phillips Tract Natural Area of the University of Illinois at Urbana-Champaign (UIUC; permission granted by J Ellis, UIUC Natural Area Coordinator) in Champaign County, Illinois, USA. At this location, the Saline Branch drains approximately 121 km^2^ and upstream land cover is primarily agriculture with some urban area and sparse forest. Additionally, this site has a United States Geological Survey (USGS) flow gage (USGS gage #03337570) that allowed us to measure discharge during our experiment. Importantly, there have been no records of *P. clarkii* in Champaign County (Taylor & Tucker, 2005), and consequently there should not have been any background eDNA at our study stream to confound our results.

### Experimental set-up

Crayfish used in this study were collected from the North Branch of the Chicago River by baited trapping during the summer of 2018, frozen at −20 °C, and then transported to UIUC where they remained in a −20 °C freezer until use. On 14 September 2018, we allocated three frozen crayfish to each stream enclosure for our experiment. First, we measured crayfish to size match them between enclosures (range: 47.2−61.3 mm total carapace length). Then, we weighed crayfish prior to placement in enclosures (mean ± SE: 42.62 ± 1.73 g per crayfish; mean ± SE: 122.87 ± 5.77 g per enclosure). Crayfish in enclosures were contained individually within labelled polyester mesh bags (30.5 cm × 20.3 cm; irregular mesh approximately 413 µm × 341 µm × 277 µm, purifyou™, USA) to restrict decomposition to microbial action rather than effects of invertebrate or vertebrate detritivores (see discussion). Mesh bags were then placed in crayfish traps (∼43.5 cm × 17.5 cm; Frabill^®^, USA) with their openings (two at each end of ∼5.5 cm each) closed to restrict access of larger consumers to carcasses. Thus, every enclosure held three crayfish, in which each crayfish was individually contained in its own mesh bag.

**Figure 1:**
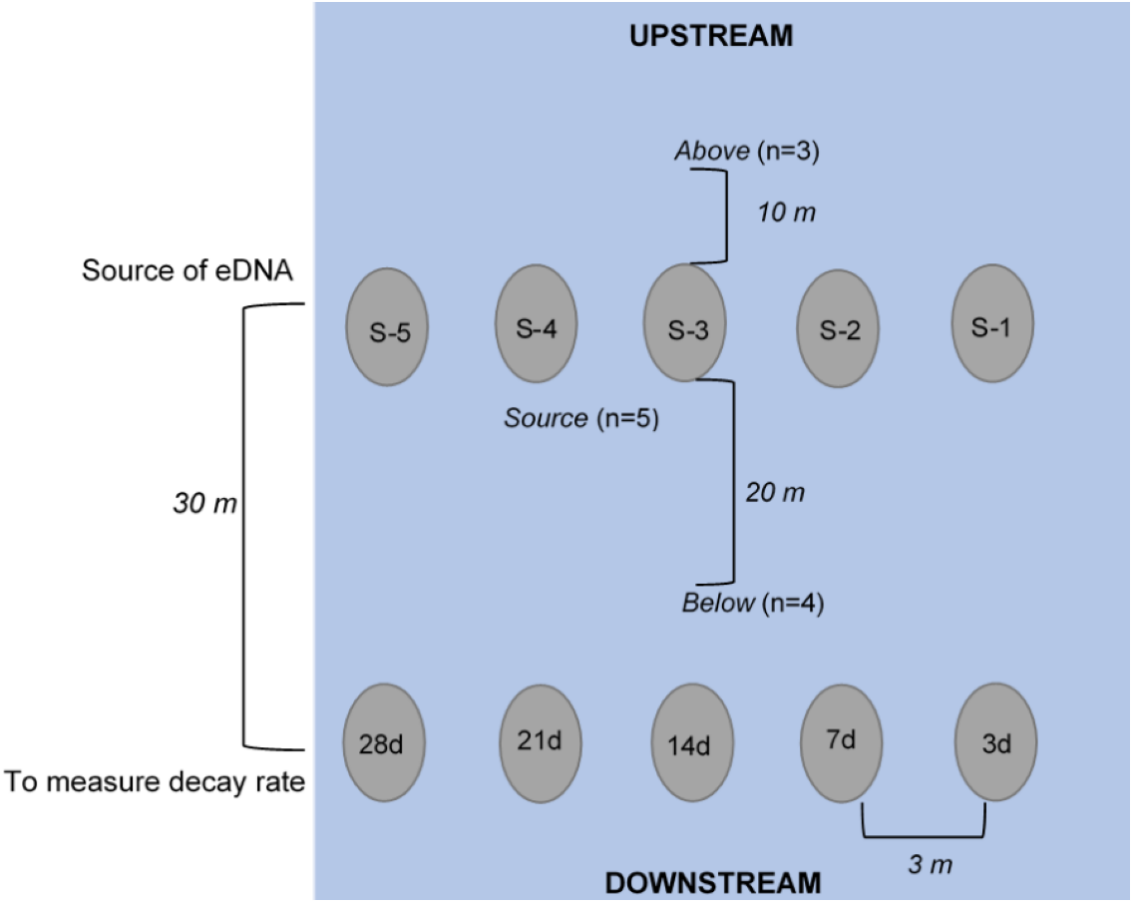
Schematic of experimental set-up (not to scale), where enclosures downstream (labeled 3d, 7d, etc.) held crayfish (*n* = 3 each) used to measure the rate of decay over the 28-day experiment. The enclosures upstream (labeled S-1, S-2, etc.) held crayfish (*n* = 3) used as the source of eDNA. Water was collected at “below” (*n* = 4, 250 mL bottles at 20 m downstream of the “enclosures”), at “source” (*n* = 5, 250 mL bottles directly in front of enclosure), and “above” (*n* = 3, 250 mL bottles at 10 m upstream of enclosures) locations. All enclosures were deployed approximately 3-m apart from one another longitudinally across the width of the stream.

Ten crayfish enclosures were attached to rebar stakes that were hammered into the streambed. Five enclosures were used as sources of eDNA and were placed 30 m upstream from the remaining five enclosures, which were used to determine the loss of crayfish biomass over time (Fig. 1). We placed all enclosures approximately 3 m apart from each other laterally across the width of the stream. The five enclosures used as eDNA sources were left in the stream for the entirety of our study and were removed after water collection on the last day. One of the five enclosures used to determine the rate of crayfish decay was removed at each of five eDNA water sampling events (days 3−28; below) to measure crayfish biomass remaining over time. After water samples for eDNA had been collected and placed in sealed bags, we removed the enclosure containing *P. clarkii* used to determine decay rate from the stream and placed the mesh bags in a plastic bag. We froze these bags at −20  °C until the conclusion of the experiment, when we then recorded wet weight of *P. clarkii* carcasses. We anticipated comparing crayfish decay rate, or estimated biomass remaining, over 28 days to eDNA copy number from our water samples.

### eDNA sampling

Water samples were collected in 250 mL clean (previously washed with 50% bleach) Nalgene^®^ (USA) bottles (Goldberg et al., 2016). Bottles were triple-rinsed on-site with stream water and then the bottle was submerged underwater until full, capped, and placed in a sealed bag. We collected water samples at day 1 (∼1.5 h after enclosure deployment), 3, 7, 14, 21, 28 after deployment, and 3 and 7 days after removal of enclosures. At each of these sampling dates, samples were collected at the following three locations: “above”, “source”, and “below” (Fig. 1). To assess whether there was background *P. clarkii* eDNA in the stream, we collected three water samples across the width of the stream approximately 10 m upstream of enclosures. Next, to examine if detectable eDNA was released from carcasses, one water sample was collected directly at the base of each of the five source enclosures (*n* = 5 per sampling date). Lastly, to assess whether carcass eDNA was transported downstream, we collected four water samples across the width of the stream 20 m below the source enclosures but 10 m above the biomass loss enclosures (Fig. 1). We expected that eDNA detections would be highest at the source enclosures, less likely downstream (below enclosures), and particularly unlikely upstream (above enclosures); therefore, we adjusted replication accordingly to save time and materials. Due to high flows (∼20,319 L/s) on 8 October 2018 (day 24), one source enclosure (S#2; Fig. 1) was displaced and lost downstream; hence the remaining eDNA samples were only collected at the remaining four source enclosures.

**Figure 2:**
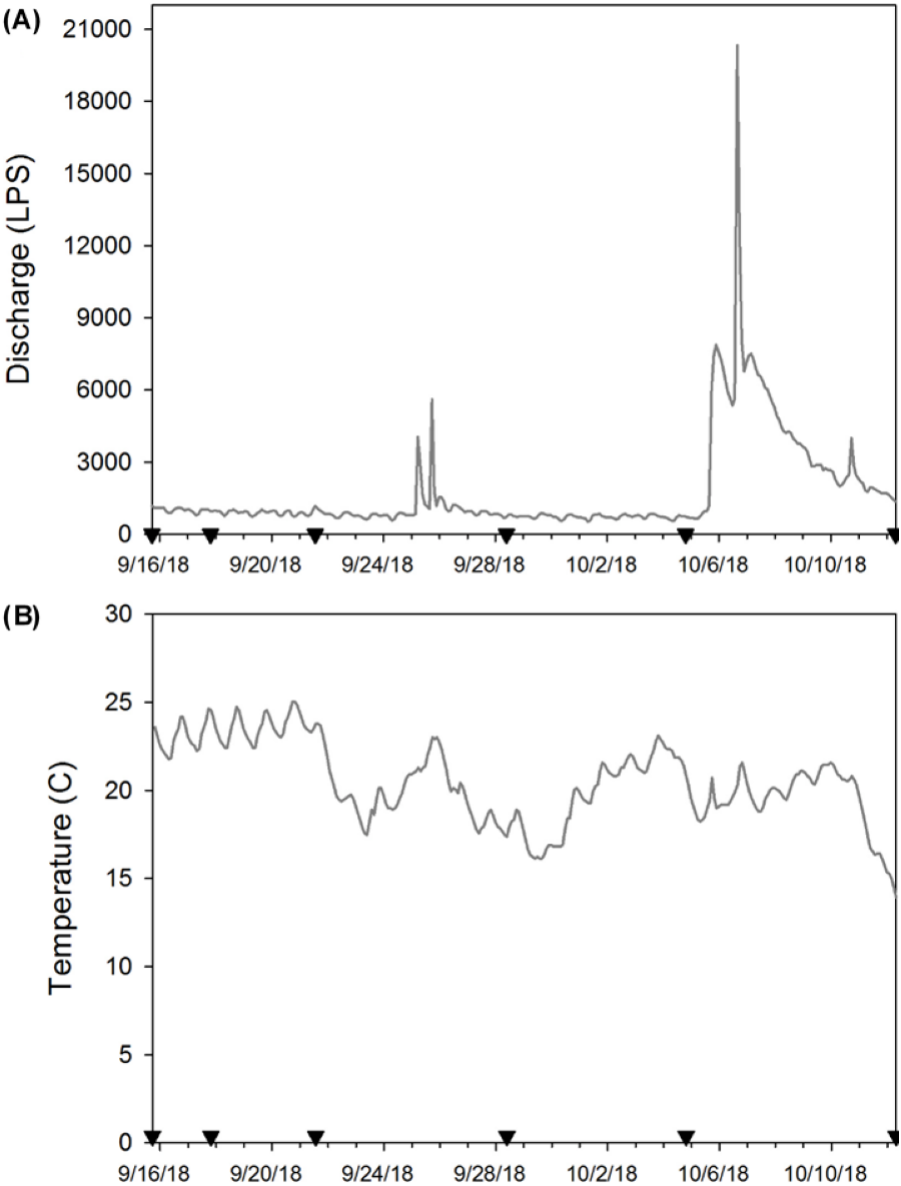
(A) Stream discharge (L/s) and (B) temperature (°C) during the enclosure experiment (9/16/18 to 10/12/18). Here the inverted black triangles indicate when eDNA samples were collected and enclosures used as the source of eDNA were removed to estimate crayfish decay. We provide stream discharge as L/s so that our results are more comparable with other published eDNA studies.

At each sampling date, two bottles (250 mL) filled with distilled water were used as field blanks to test background contamination. Field blanks were transported to the field and handled in the same way as field samples for eDNA. Additionally, on every sampling date, we recorded temperature, pH, conductivity, total dissolved solids, and salinity using a hand-held probe (Oakton^®^, USA), and turbidity was recorded with a portable meter (Sper Scientific©, USA; Table 1). To monitor temperature during our experiment, we attached a HOBO Pendant^®^ Temperature/Light 8K logger (Onset^®^, USA) to one enclosure on 15 September 2018 (the day after deployment) and logged data every two hours until day 28 when all crayfish enclosures were removed (Figs. 2A & 2B). We do not report light data here because periphyton growth on the logger affected these results. We anticipated that water chemistry (Strickler, Fremier & Goldberg, 2015), temperature (Eichmiller, Best & Sorensen, 2016), and stream flow (Jane et al., 2015; Stoeckle et al., 2017) could influence eDNA persistence and detection in our system. Thus, we provide these site characteristics to facilitate comparison of our experiment to other study systems.

**Table 1:** Water chemistry values during the experiment from carcass deployment (day 0) to removal (day 28) to one-week post-removal.

**Sampling Point (day)**	**Salinity (PSU)**	**pH**	**TDS(mg/L)**	**Conductivity (µS/cm)**	**Turbidity (NTU)**
0 (Deployment)	0.48	8.53	657	926	0.16
3	0.48	8.43	654	922	0
7	0.49	8.42	654	923	5.34
14	0.52	8.42	710	1,000	0.97
21	0.56	8.30	763	1,077	4.67
28 (Removal)	0.44	8.61	613	864	6.32
3-post removal	0.44	8.64	604	851	4.70
7-post removal	0.46	8.74	651	890	0.64

After all eDNA samples were collected on a date, each enclosure was cleared of debris (garbage, leaf litter, etc.) to ensure water flow was similar across all enclosures. Water samples were placed on ice in a cooler and transported back to UIUC, where they were filtered within two hours of collection. To avoid carry-over contamination from handling *P. clarkii* carcasses, we showered and changed clothes prior to filtration. In addition, we wore disposable nitrile gloves and used frequent glove changes during all field collection and subsequent lab work (e.g., filtration, extraction, and qPCR).

Prior to filtration, the entire work surface was cleaned with a 50% bleach solution and all materials used (e.g., funnels, forceps) had been previously washed with a 50% bleach solution (Goldberg et al., 2016) and subsequently rinsed with deionized water. To minimize any potential contamination during filtration, we filtered water samples in the following order: field blanks, above, below, and source samples. Water samples were vacuum filtered onto 1.0 µm cellulose nitrate filters (Whatman™, General Electric Healthcare, USA) and then placed into two mL centrifuge tubes filled with one mL of cetyl trimethylammonium bromide (CTAB; Teknova©, USA). Filters remained in the dark at room temperature (∼20−22 °C) for approximately two months, to reduce degradation by UV-B, to increase cell lysis over time (Renshaw et al., 2015; Wegleitner et al., 2015), and so that all filters would be in CTAB for the same length of time and be handled in the same manner. After two months, filters in CTAB were frozen at −80  °C until extraction. We then extracted DNA using a chloroform isoamyl alcohol extraction procedure (Renshaw et al., 2015), which can increase DNA yield relative to other eDNA methods and is resistant to many common PCR inhibitors (Schrader et al., 2012). One extraction blank for every ∼12 eDNA samples was used to test the level of lab contamination.

### qPCR assay

We used the *P. clarkii* primer-probe assay developed by Tréguier et al. (2014) to amplify a 65 bp fragment of the COI region:

F-primer: 5′-AACTAGGGGTATAGTTGAGAG-3′

R-primer: 5′-CAGAAGCTAAAGGAGGATAA-3′

Probe: 5′-FAM-AGGAGTTGGAACAGGATGGACT-MBG-3′.

We conducted initial assay optimization by running different primer and probe concentrations and modifying the annealing temperature and selected the conditions that produced the best results (e.g., earliest Cq values, highest *R*^2^ values, and greatest % efficiency; Fig. S1). A 20 µL qPCR reaction was run on a QuantStudio 3 Real-Time PCR (Applied Biosystems^®^, USA) composed of: 10 µL iTaq™ Universal Probes Supermix (Bio-Rad Laboratories, USA), 5.2 µL of sterile water, 0.8 µL of each primer (10 µm), 0.2 µL probe (10 µm), and 3 µL of eDNA. The following qPCR procedure was used to amplify DNA: initial denaturation at 50 °C for 5 min and 95 °C for 10 min, and then 95 °C for 30 s and 60 °C for 1 min for 40 cycles (adapted from Tréguier et al., 2014).

In a clean room, we prepared all plates, added all eDNA samples, and then transported sealed strip tubes to our physically separate qPCR lab and added standards. All samples were run in triplicate with negative controls (3 µL of the master mix replaced DNA). Using *P. clarkii* from the same source as our enclosure experiment, we extracted DNA from muscle tissue using a DNeasy^®^ Tissue and Blood Kit (Qiagen©, Germany). We amplified this DNA using the primer-probe assay developed by Tréguier et al. (2014) to concentrate a 65 bp gene fragment of only *P. clarkii* DNA. Then we quantified this DNA (Qubit^®^ Fluorometer, ThermoFisher, USA) and used it to run standards (1:10 dilution) from 1. 4 ×10^7^ copies/µL (1 × 10^−3^ ng/µL gene fragment) to 1.4 copies/µL (1 × 10^−10^ ng/µL gene fragment) on each plate and to create a standard curve. All standard curve analyses (*R*^2^, % efficiency, slope, etc.) and Cq values were calculated using the Thermo Fisher Connect™ Cloud (Applied Biosystems^®^, USA) interface. Here we define our limit of detection (LOD) as the lowest concentration standard that amplified in 1/3 wells (Hunter et al., 2016) and our limit of quantification (LOQ) as the lowest concentration standard that always produced replication in 3/3 wells (Tréguier et al., 2014). Context on *P. clarkii* COI mtDNA haplotypes present at our Chicago River collection site is provided in Oficialdegui et al. (2019).

### Crayfish biomass loss over time

At the conclusion of the experiment, frozen crayfish were rinsed of sediment and debris, patted dry, and then weighed (± 0.01 g; Proster^®^, United Kingdom) to obtain a final wet weight. Then to calculate the loss of biomass over time for each individual, we took the difference between the initial weight (before deployment) and the weight after removal. We did this for the crayfish enclosures that were removed on day 3, 7, 14, 21, 28, as well as for the crayfish used in the eDNA source enclosures upon their removal.

### Statistical analyses

We fit a Michaelis–Menten curve to the loss in *P. clarkii* biomass over time, from the biomass enclosures removed at each sampling date, using the *drc* package (Ritz et al., 2015) in R (v. 3.6.2). We used a Michaelis–Menten model to represent a plateau in which crayfish carcass decay slowed in our experiment after soft tissues had been lost but the exoskeleton of the carapace or chelae remained. In addition, we compared the initial mass of the individual crayfish used in the source enclosures to the final mass at the conclusion of the experiment using a paired *t*-test. Data met parametric assumptions and no transformations were used. Lastly, we anticipated using multiple linear regression models with AIC model competition (Burnham & Anderson, 2004) to evaluate whether crayfish biomass, discharge, temperature, or other water chemistry variables explained eDNA copy number and detectability over time.

### Sensitivity tests of results

Given our initial results, we subsequently sought to examine whether some non-detections of *P. clarkii* eDNA from carcasses could be attributable to either poor performance of the Tréguier et al. (2014) assay across intraspecific genetic diversity within our species (Oficialdegui et al., 2019) or PCR inhibition of our field samples (Schrader et al., 2012). First, we collected nine additional *P. clarkii* individuals from the North Branch of the Chicago River in August 2019**,** and then extracted DNA from muscle tissue using a DNeasy^®^ Tissue and Blood Kit (Qiagen©, Germany). Then we used a random number generator to select eDNA water samples from our preceding experiment. To these eDNA water samples, we followed the same procedure as above and added a 1:10 serial dilution from 1. 4 ×10^7^ copies/µL (1 × 10^−3^ ng/µL gene fragment) to 1.4 copies/µL (1 × 10^−10^ ng/µL gene fragment) using the nine additional crayfish and DNA from the individual *P. clarkii* originally used to create standard curves (10 total). This allowed us to examine if samples across more *P. clarkii* individuals reliably amplified and if amplifications were delayed (e.g., inhibited) relative to the original serial dilution series from assay testing. We followed the same qPCR recipe as above but added 3 µL of extracted *P. clarkii* serial dilution to each well and ran plates following the same qPCR procedure.

### Confirmation of DNA in samples

To ensure that non-detections of *P. clarkii* carcass eDNA were not due to methodological decisions (e.g., CTAB storage, chloroform extraction) and that eDNA was present in our samples, we first measured the concentration of all samples on a Qubit™ 2.0 using a dsDNA HS Assay Kit (Invitrogen, U.S.A). We then ran a subset of eDNA samples (those collected on days 0, 3, and 7) on qPCR for the invasive Asian clam (*Corbicula fluminea*) common in our study stream (∼6.8 *C. fluminea*/m^2^; A Curtis, 2018, unpublished data). We used an existing *C. fluminea* eDNA assay (Cowart et al., 2018b) with a new genus-specific probe to amplify a 208 bp COI fragment:

F-primer: 5′-TTTATTAGATGATGGGCAGCTGTA-3′

R-primer: 5′-TGATCTAACCAACAAAAGCATAGC-3′

Probe: 5′-FAM-AGTGATGCCAATAATAATGGGTGGTTTTGG-MGB-NFQ -3′.

All samples were prepared in a dedicated clean room using the following: 10 µL iTaq™ Universal Probes Supermix (Bio-Rad Laboratories, USA), 6.15 µL of sterile water, 0.35 µL of each primer (10 µm), 0.15 µL probe (10 µm), and 3 µL of eDNA. Samples were run in triplicate, including non-target controls on each plate on a QuantStudio 3 Real-Time PCR (Applied Biosystems^®^, Foster City, CA) with the following parameters: 95 °C for 10 min followed by 40 cycles at 95 °C for 15 s and 62 °C for 1 min. We used a gBlock fragment (Integrated DNA Technologies, USA) to run serial dilutions (1:10) from 4. 4 × 10^6^ copies/µL (1 × 10^−3^ ng/µL) to 0.44 copies/µL (1 × 10^−10^ ng/µL), and to generate standard curves for both plates (Cowart et al., 2018b).

## Results

**Figure 3:**
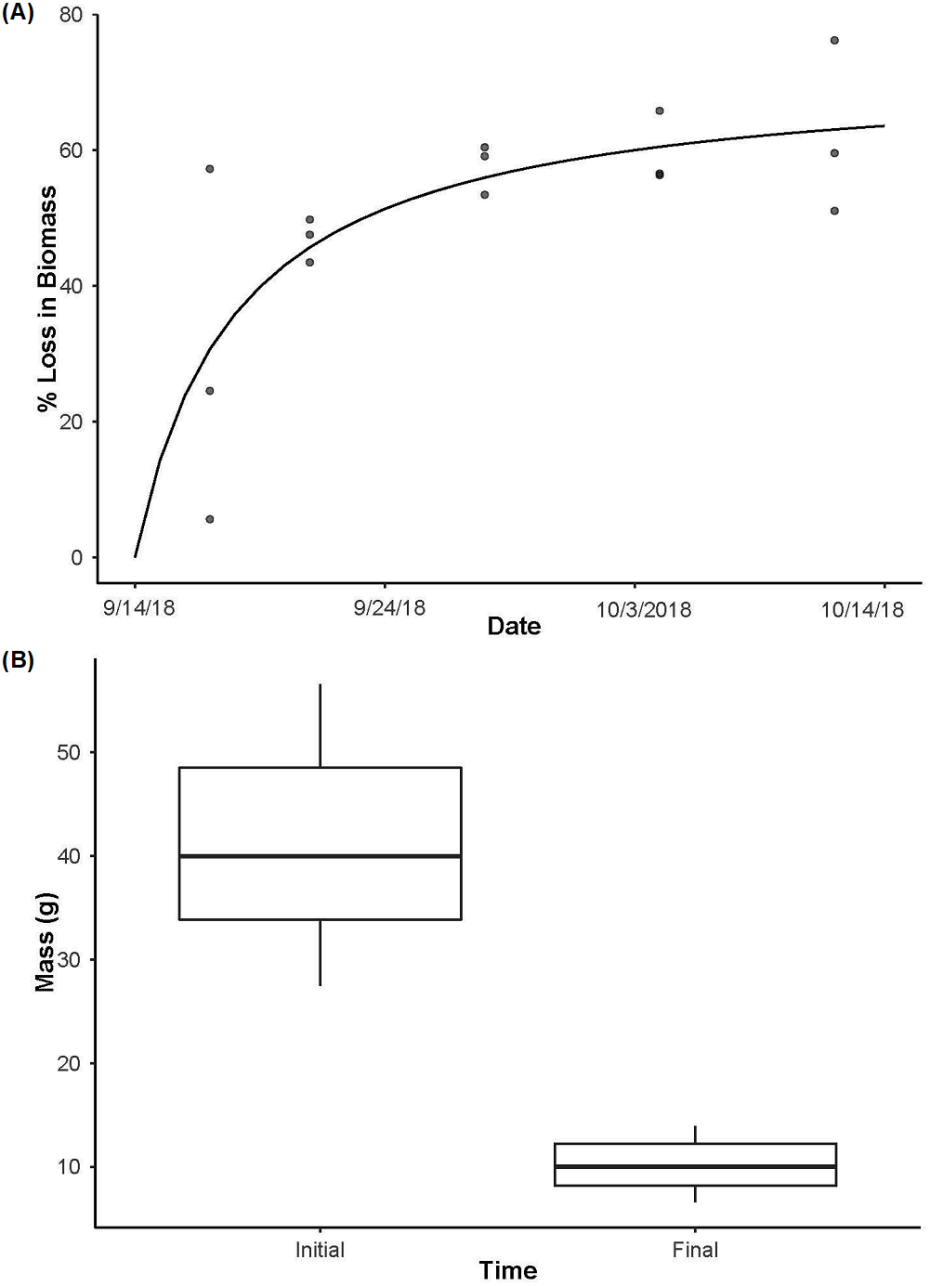
Crayfish decay. (A) The loss in biomass (wet weight loss) over time from day 3 to day 28 with a Michaelis-Menten model fit (*R*^2^ = 0.85, *P* < 0.001) to the data for crayfish removed from decay cages. (B) The initial mass of individual crayfish used as the source of eDNA (*n* = 15) and then the final mass of crayfish after being deployed for 28 days (*n* = 12, as one enclosure was displaced and lost during high flows) highlighting the loss of crayfish biomass over time (*t*_11_ = 14.32, *P* < 0.001). Boxplots represent the median value and interquartile range, and whiskers indicate the minimum and maximum values.

In the biomass enclosures, *P. clarkii* biomass declined over time (Fig. 3A; *R*^2^ = 0.85, *P* < 0.001), from an average of ∼71% biomass remaining on day 3 to a plateau of ∼38% remaining on day 28 (Fig. 3A). Further, in the source enclosures that were deployed for the full 28 days, total crayfish biomass was significantly lower at the conclusion of the experiment relative to the start (Fig. 3B, *t* = 14.32, *df* = 11, *P* < 0.001). However, even at day 28 much of the crayfish carapace and chelae remained. More specifically, the total initial biomass of the source cages was 614.7 g (mean ± SE: 122.93 ± 8.99 g per cage) and 98.67 g (mean ± SE: 31.04 ±  2.26 g per cage) remained on day 28 (one enclosure holding three crayfish was lost due to high flows and thus the final biomass estimate is lower than might be expected). On average, crayfish from the eDNA source enclosures lost ∼74% of biomass over the 28-day deployment.

**Figure 4:**
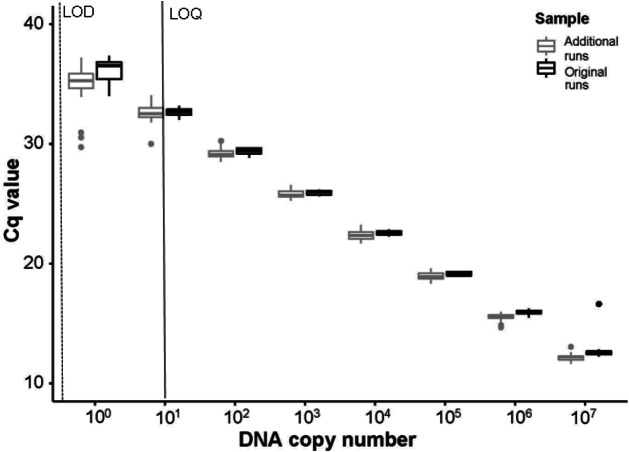
Comparisons of Cq values between initial and additional qPCR runs. The Cq values from serial dilutions (10^7^–10^0^ DNA copy number or 1 × 10^−3^ ng/μ L –1 × 10^−10^ ng/μ L gene fragment) run on the initial experimental plates, denoted as “Original runs.” In comparison, the Cq values from the combination of serial dilutions run using extracted DNA from ten *P. clarkii* (nine additional crayfish and the initial DNA used to create the standard curves in our experimental plates) with randomly selected eDNA samples, denoted as “Additional runs.” Here we show that the Tréguier et al. (2014) assay amplified intraspecific genetic variation in *P. clarkii* collected from the North Branch of the Chicago River, USA and that non-detections in eDNA samples were not caused by inhibition. From the initial standard curve, LOD here is shown in a dashed line at ∼0.14 copies/μ L (1 ×10^−11^ ng/ μ L gene fragment); LOQ is signified with a black line at ∼14 copies/μ L (1 ×10^−9^ ng/μ L gene fragment). Boxplots show the median and interquartile ranges, whiskers show the highest and lowest values without outliers, and points extending past whisker denote outliers.

No field blanks, extraction blanks, or negative plate controls amplified. From the standard curves, run using the serial dilutions of *P. clarkii* DNA on every plate, slopes ranged between −3.317 and −3.447, *y*-intercepts ranged between 34.010−37.344, *R*^2^ values were between 0.997−1.000, and assay efficiency ranged between 95−103%. Our LOD was 0.14 copies/µL (1 ×10^−11^ ng/µL gene fragment) and our LOQ was 14 copies/µL (1 ×10^−9^ ng/µL gene fragment). In our sensitivity tests following enclosure experiment results, the assay successfully amplified DNA from all ten *P. clarkii* individuals used without apparent differences in assay performance by crayfish. We found no evidence that our samples were inhibited, as curves from the serial dilutions added into field eDNA samples amplified at similar or earlier Cq values relative to the serial dilutions run on the initial plates (Fig. 4). Therefore, the assay used here worked well and amplified *P. clarkii* tissue from crayfish that were collected at the same location as those used in our experiment and without evidence of inhibition. However, none of our field eDNA samples amplified from any of the sample locations or dates, indicating that crayfish carcasses were not producing detectable eDNA in this system.

Quantification of our eDNA samples indicated that DNA was present (Data S1). When a subset of eDNA samples were run on qPCR for *C. fluminea*, none of the field blanks, extraction blanks, or negative plate controls amplified. From the standard curves, the slopes ranged between −3.426 to −3.474, y-intercepts ranged between 35.416−36.461, R^2^ values were between 0.996−0.999, and assay efficiency was 94−95.8% (Data S1). Our LOD was ∼0.44 copies/µL (1 × 10^−10^ ng/µL) and our LOQ was ∼4.4 copies/µL (1 × 10^−9^ ng/µL). All samples amplified in at least 1/3 wells and most amplified in 3/3 wells (Data S1), further indicating that methodological choices cannot account for lack of amplification of *P. clarkii* eDNA.

## Discussion

Here we demonstrated that crayfish carcasses did not release detectable eDNA at the biomass used (15 *P. clarkii*) or under the abiotic conditions (temperature, flow, pH) of this stream enclosure experiment. Although research on this topic has been limited, some past studies have found carcasses can produce detectable eDNA (Merkes et al., 2014; Dunker et al., 2016; Kamoroff & Goldberg, 2018; Tillotson et al., 2018; Strand, 2019). However, these previous studies have largely been restricted to lab experiments (Merkes et al., 2014), mesocosms (Kamoroff & Goldberg, 2018), or in field locations (Strand, 2019) where living target individuals may confound results (Dunker et al., 2016) or have recently spawned (Tillotson et al., 2018). We speculate that our results differ from previous research largely due to differences in system-specific conditions (lentic vs. lotic and lab vs. in situ) and taxa.

Due to the chitinous exoskeleton of crayfish and their benthic habitat use, some researchers have suggested that crayfish eDNA may be more difficult to detect than other taxa that occur in the water column or shed more mucous, like fish or amphibians (Tréguier et al., 2014; Rice, Larson & Taylor, 2018). However, successful detection of low abundances of crayfish using eDNA has been documented (Dougherty et al., 2016; Cai et al., 2017). In the original *P. clarkii* eDNA study, Tréguier et al. (2014) were successful in detecting *P. clarkii* in 57 out of the 78 ponds known to have *P. clarkii* present (∼73% success rate) using eDNA. Tréguier et al. (2014) conducted their research in lentic environments, collected smaller sample volumes, and used different laboratory methods relative to our study. Two additional *P. clarkii* assays (Geerts et al., 2018; Mauvisseau et al., 2018) have been developed and implemented since Tréguier et al. (2014), and both were reported to possibly work better to amplify *P. clarkii* eDNA. However, Geerts et al. (2018) only used the primers developed by Tréguier et al. (2014) and may have decreased the specificity and success of the assay by neglecting to include the probe. Mauvisseau et al. (2018) found that the initial assay (Tréguier et al., 2014) could detect *P. clarkii* eDNA, but did not work well using TaqMan™ master mix. Here we demonstrated that the Tréguier et al. (2014) assay was excellent (good efficiency and low LOD) in amplifying tissue extracted from 10 *P. clarkii* individuals collected from the North Branch of the Chicago River, USA. In addition, others have found the assay developed by Tréguier et al. (2014) to be successful (100% success rate) in detecting *P. clarkii* eDNA from water samples (Cai et al., 2017). Per our LOD estimates, we should have been able to detect eDNA from *P. clarkii* down to ∼0.14 copies/ µL (10^−11^ ng/µL gene fragment), yet we were unable to do so from carcasses.

The *P. clarkii* biomass used here might have been too low relative to stream discharge to produce detectable eDNA. However, eDNA can detect organisms with low abundance and low biomass (e.g., Goldberg et al., 2013; Dougherty et al., 2016; Cowart et al., 2018a). For example, Cai et al. (2017) were able to detect eDNA at ∼1 live *P. clarkii* in 100,000 L of water compared to the 15 crayfish in ∼515 L (∼1 crayfish/34 L) at the lowest flow and ∼20,319 L (1 crayfish/ 1,355 L) at the highest flow used in this experiment. Additionally, the biomass used in this experiment (initially ∼616 g or ∼1.2 g/L) is comparable to the 1 g/L used by Dunn et al. (2017) that produced detectable eDNA from signal crayfish (*Pacifastacus leniusculus*) in a laboratory setting. Moreover, the sample volume or replication used here might have been insufficient to detect eDNA. Previous research has demonstrated that increasing sample volume and the number of qPCR replicates can improve detection (e.g., Harper et al., 2018; Doi et al., 2019; Sepulveda et al., 2019). However, other in situ eDNA studies have used similar or smaller volumes of water and sample replicates and were able to detect crayfish at low abundances (e.g., Dougherty et al., 2016; Rice, Larson & Taylor, 2018; Robinson et al., 2018) and our use of 250 mL sample volumes was sufficient to amplify *C. fluminea* eDNA in these samples. Therefore, we believe that the biomass, sample volume, and number of replicates used in our study should have resulted in detectable eDNA, and we conclude that crayfish carcasses likely produce less eDNA than living organisms of this taxa.

Crayfish biomass loss occurred rapidly, with only ∼26% of the biomass remaining at the end of the experiment in our source enclosures. As such, crayfish tissue and DNA were being released into the stream, yet we did not detect eDNA from our sampling. Others have reported that open (i.e., not enclosed) fish carcasses experience rapid decay with complete biomass loss in as few as four days in a stream (Minshall, Hitchcock & Barnes, 1991), but Elliott (1997) reported ∼45% biomass loss after 20 days when carcasses were enclosed (protected from scavengers). If the crayfish used here were not contained in mesh bags within the enclosures, it is plausible that no or minimal biomass would remain at the end of the experiment due to consumption of the carcasses by vertebrate or invertebrate scavengers. Notably, both live crayfish and fish were often found in our enclosures (mesh bags remained closed), despite our attempts to close the entrances of these crayfish traps. Further, crayfish carcasses would likely have been transported downstream, particularly during high flows, thereby further decreasing the likelihood of eDNA detection at our sampling site. Therefore, our enclosure of carcasses in mesh bags likely resulted in slower decay rates that should have made eDNA persist in the stream for longer. Accordingly, we expect that our experimental design should have increased the likelihood of detecting eDNA from crayfish carcasses relative to more natural conditions.

A variety of choices made during any eDNA study can ultimately affect the results (e.g., Goldberg et al., 2016; Tsuji et al., 2019). Despite our attempts to control for a number of these factors, various abiotic (i.e., UV-B, inhibition) or biotic (i.e., use of frozen carcasses) conditions may have influenced our study. For example, UV-B radiation can reduce the persistence of eDNA (Strickler, Fremier & Goldberg, 2015) and was not measured in our study, although our enclosures were well-shaded by a dense riparian forest canopy. Additionally, UV-B radiation may alter long-term eDNA persistence and detectability (Strickler, Fremier & Goldberg, 2015), but it should not have influenced our samples collected immediately following enclosure deployment. Next, selection of filter or extraction methods may have impacted our results. Our use of 1.0 µm filters was consistent with past results that have found 0.4 µm to 1.2 µm filters to work well for carcass eDNA relative to larger pore sizes (Kamoroff & Goldberg, 2018). Inhibition often caused by tannins and humic acids can reduce the potential of qPCR reactions and the overall ability to detect eDNA (i.e., increase false negatives). However, the combined used of CTAB and subsequent chloroform isoamyl alcohol DNA extraction procedure has been repeatedly documented to reduce inhibition and result in higher DNA yields than other eDNA methods (e.g., Schrader et al., 2012; Renshaw et al., 2015; Williams, Huyvaert & Piaggio, 2017; Hunter et al., 2019). Here we directly assessed whether inhibition in our eDNA water samples may have affected our results. We re-ran our eDNA samples with serial dilutions of *P. clarkii* DNA added and then compared the Cq values to those estimated from our original standard curves. The Cq values were similar across the dilution series regardless of whether they were in environmental samples or not, showing no shift to delayed Cq values associated with PCR inhibition.

Additionally, it seems unlikely that our decision to preserve filters in CTAB at room temperature and then use a chloroform extraction protocol degraded DNA and resulted in non-detections. Filters stored in CTAB at room temperature produce more eDNA (due to cell lysis) than CTAB filters stored in a −20 °C freezer, and this protocol produces degrees of magnitude more eDNA than other methods like ethanol preservation followed by extraction with Qiagen kits (Renshaw et al., 2015; Tsuji et al., 2019). All of our field samples amplified in at least 1/3 of plate replicates when we re-ran a subset for a different species that occurs at our study site (*C. fluminea*), indicating that our storage and extraction protocols do not explain non-detections of eDNA from *P. clarkii* carcasses. In addition, past work by our laboratory using this method to detect rare endangered and invasive organisms (e.g., de Souza et al., 2016; Cowart et al., 2018a; Cowart et al., 2018b; Kessler et al., 2020) further demonstrates its consistent sensitivity to low abundances of eDNA. Thus, we conclude that it is unlikely that our non-detections can be attributed to effects of UV-B, inhibition, or methodological decisions around sample handling and extraction.

The decision to use frozen carcasses was a byproduct of ease in storage and transportation from the Chicago River to UIUC and could have reduced or delayed natural microbial decomposition, affecting our results. For example, Micozzi (1986) noted that in the short-term (days) aerobic decomposition drove the terrestrial decay of frozen lab rats (*Rattus norvegicus domestica*), while fresh rat carcasses had largely anaerobic decomposition. Information on decay and decomposition in aquatic systems is somewhat limited (Elliott, 1997; Minshall, Hitchcock & Barnes, 1991), and we are not aware of any published studies that have directly examined crayfish decay to compare with our results. Nevertheless, other comparable eDNA studies have used frozen carcasses and reported detectable levels of eDNA (Merkes et al., 2014; Dunker et al., 2016). In summary, we acknowledge that a number of unmeasured factors may have contributed to our inability to detect eDNA but conclude that crayfish carcasses at low densities in lotic systems do not produce detectable eDNA. Further, we speculate that our non-detections are likely due to the combined dilution and transport of eDNA in a lotic system coupled with the overall lower concentrations of eDNA released by carcasses compared to live organisms.

Our results are encouraging for the use of eDNA to monitor outcomes of management actions for either invasive or imperiled crayfish and potentially other taxa. Eradication efforts for invasive crayfishes are common (e.g., Freeman et al., 2010; Gherardi et al., 2011; Hansen et al., 2013; Manfrin et al., 2019), but eDNA could produce misleading results if positive detections are produced by carcasses that remain after the management effort (Dunker et al., 2016; Kamoroff & Goldberg, 2018; Carim et al., 2020). These false positives could be costly if they prompt further eradication or monitoring that is not warranted (Darling & Mahon, 2011; Jerde et al., 2013; Carim et al., 2020). Similarly, reintroduction efforts of endangered species could be inaccurately interpreted as successful if eDNA from a species is detected only from carcasses and not from living individuals (Laramie, Pilliod & Goldberg, 2015; Cowart et al., 2018a). Our study suggests that when crayfish biomass is low, eDNA detections from carcasses are unlikely. However, we caution that management actions should not be based on single or short-term eDNA results and recommend that other more traditional methods or repeated long-term eDNA sampling be used to confirm initial eDNA results (Darling & Mahon, 2011; Dunker et al., 2016; Cowart et al., 2018a). We propose that future studies replicate our work in-situ in other ecosystems examining additional taxonomic groups. In addition, future research should examine the effects of field sample volume and replication on detection probabilities for eDNA from carcasses (Doi et al., 2019). Further, laboratory, mesocosm, or in situ experiments that compare eDNA production and detectability between live organisms and carcasses would be useful. Future studies might also examine the effect biomass, stream size, and abiotic conditions, such as temperature or seasonality, on carcass eDNA.

## Conclusion

Positive eDNA detections were not produced by *P. clarkii* carcasses enclosed in our study stream during the 28-day experiment or seven days after carcasses were removed from the stream. Therefore, our results suggest that eDNA detections from carcasses are unlikely when crayfish are rare, such as in cases of new invasive populations or endangered species, and that positive detections may indicate the presence of live organisms. However, future studies are necessary to examine how biomass, flow, and differences in system (lentic vs. lotic) or taxa (fish vs. invertebrate) influence the ability to detect eDNA from carcasses.

##  Supplemental Information

10.7717/peerj.9333/supp-1 Figure S1 Optimization steps used to adapt the Tréguier et al., 2014 P. clarkii eDNA assay for our study

10.7717/peerj.9333/supp-2 Supplemental Information 1 Qubit data and additional runs for *C. fluminea* eDNA Qubit concentrations and results from two qPCR runs of *P. clarkii* eDNA samples run for another species (*Corbicula fluminea*)

10.7717/peerj.9333/supp-3 Supplemental Information 2 Data used in Figs. 2A & 2B

10.7717/peerj.9333/supp-4 Supplemental Information 3 Data used to create Fig. 3A

10.7717/peerj.9333/supp-5 Supplemental Information 4 Data used to create Fig. 3B

10.7717/peerj.9333/supp-6 Supplemental Information 5 Data used to estimate biomass loss in source enclosures

10.7717/peerj.9333/supp-7 Supplemental Information 6 Data used to estimate biomass loss

10.7717/peerj.9333/supp-8 Supplemental Information 7 Data used to create Fig. 4

10.7717/peerj.9333/supp-9 Supplemental Information 8 R code file

## Additional Information and Declarations

### Data Availability

The following information was supplied regarding data availability: All associated data and the R code are available as Supplementary Files.
