# Supplementary figures and images for: No evidence that crayfish carcasses produce detectable environmental DNA (eDNA) in a stream enclosure experiment
Source: PeerJ. 2020 Jun 11;8:e9333. doi: 10.7717/peerj.9333 (PMC7293857; doi:10.7717/peerj.9333)

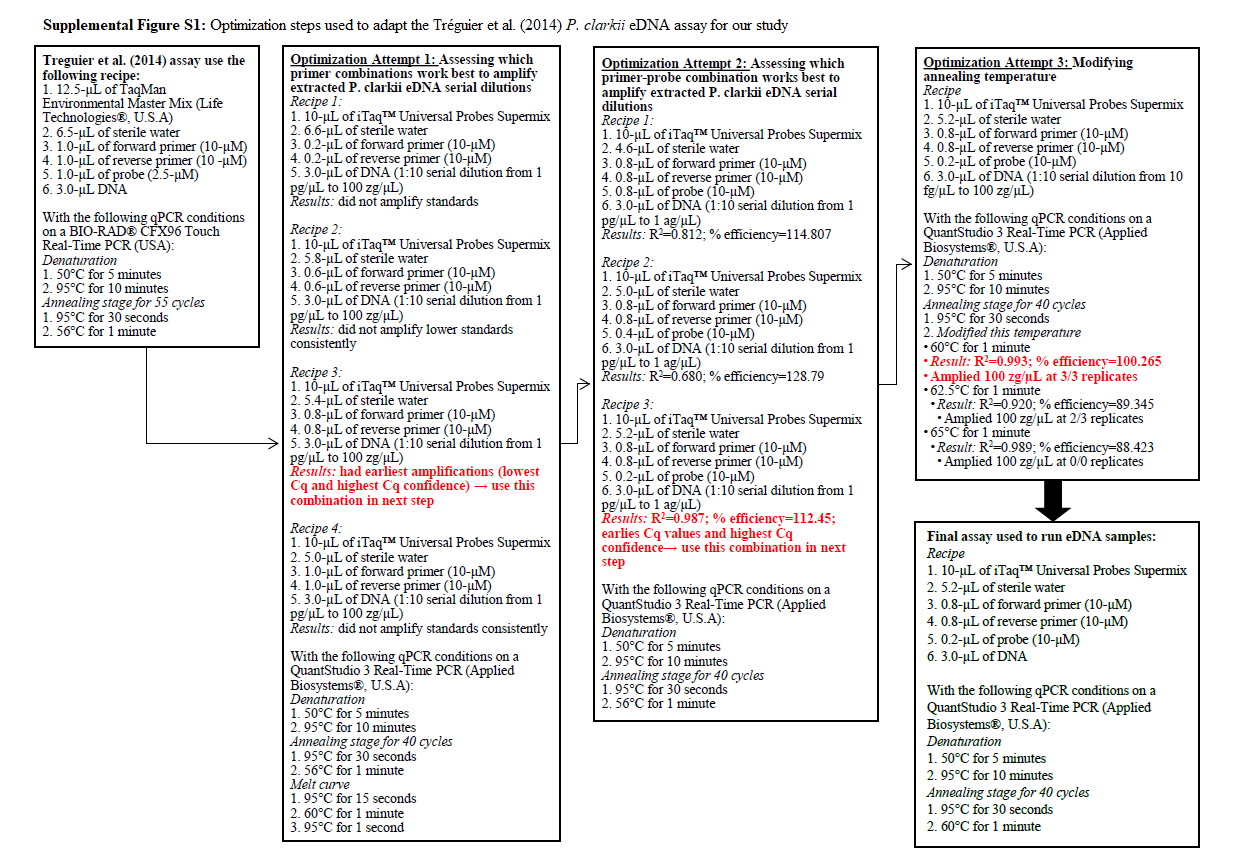

Supplement: Figure S1 [file peerj-08-9333-s001.png]
